# Domestic Summary

**Published:** 1839-01-12

**Authors:** 


### DOMESTIC SUMMARY.

In consequence of communications between members of the American Philosophical Society, in Philadelphia, and gentlemen in Boston, a meeting was held in the latter place, of gentlemen belonging to Boston, Salem, and the University at Cambridge, at which the proceedings were as follows:

His Excellency, Governor Everett, was requested to take the chair.

The Hon. Francis C. Gray was chosen Secretary.

The Chairman stated the objects of the meeting.

Dr. Warren offered and explained the three following resolutions, which were eloquently supported by the Hon. Judge Story and other gentlemen, and unanimously adopted.

1. *Resolved*, That it is expedient to form an Institution to be called the AMERICAN INSTITUTION FOR THE CULTIVATION OF SCIENCE, having for its object the advancement of physical science and literature, by assembling those interested in this object at stated periods, thus effecting an interchange of discoveries and improvements between the inhabitants of different parts of the country.

2. *Resolved*, That the organization of such an Association can best be accomplished by scientific and literary persons situated in a central part of the country, and that therefore we recommend that the American Philosophical Society, in Philadelphia, be invited to undertake this organization, with the understanding that the meetings be held successively in the different great cities of the Union.

3. *Resolved*, That as frequent meetings of those here assembled might not be practicable, a Committee of Correspondence be created, whose duty it shall be to call meetings when necessary, to communicate with the American Philosophical Society, and other scientific associations, and to advance the object of this meeting by all means in their power.

*Committee.*—Dr. Warren, Gov. Everett, Hon. Judge Story, John Pickering, Esq., Hon. F. C. Gray, Daniel Treadwell, Esq., Dr. Hale.

